# Walking Exercise and Its Relationship to Serum Lipids in Japanese

**DOI:** 10.2188/jea.12.64

**Published:** 2007-11-30

**Authors:** Yoshihisa Fujino, Tetsuya Mizoue, Noritaka Tokui, Takesumi Yoshimura

**Affiliations:** 1 Institute of Industrial Ecological Sciences, University of Occupational and Environmental Health, Japan.

**Keywords:** cross-sectional studies, lipids, walking

## Abstract

This study sought to investigate the effects of walking on serum lipids among the middle-aged and elderly. The subject group included 3312 adult Japanese who underwent a routine health examination at Yukuhashi city, Fukuoka Prefecture, in 1998. The amount of walking in which the subjects engaged and other lifestyle characteristics were examined by a self-reported questionnaire. Analyses of variance were performed to calculate adjusted means of total cholesterol, HDL cholesterol, triglyceride, and LDL cholesterol using walking time as the level of a factor. Multiple logistic regression analyses were also performed to estimate odds ratios and 95% confidence intervals for unfavorable lipid profiles. For both sexes, the adjusted mean for total and LDL cholesterol was higher in individuals who walked than in those who did not walk, and also individuals who walked had higher odds ratios for higher total cholesterol levels than those who did not walk. For men, the adjusted mean for HDL cholesterol was higher in individuals who walked than in those who did not walk. No significant difference was observed in triglyceride or the ratio of total cholesterol to HDL cholesterol between individuals who walked and those who did not walk. This suggests that walking exercise may not achieve the beneficial effect on lipids profiles among middle-aged/older Japanese.

## INTRODUCTION

Physical activity has been shown to be beneficial to physical and mental health^1^^-^^4^^)^. Recent studies have shown that walking, as compared with vigorous exercise, is also beneficial to health^5^^-^^9^^)^. In particular, walking is strongly associated with reduced risk of coronary heart disease^5^^,^^6^^)^. The specific mechanisms of this effect remain unclear, but it is believed that improving the blood lipid profile may be one of the responsible factors^10^^,^^11^^)^. Many studies have shown that walking increases the level of high-density lipoprotein (HDL) cholesterol^8^^,^^9^^,^^12^^-^^15^^)^, although the association between walking and total cholesterol, a strong risk factor for coronary heart disease, is inconsistent^8^^,^^12^^-^^15^^)^.

As a general rule, any individual with hypercholesterolemia is recommended to engage in walking for exercise. However, the specific effect of walking, the most common form of exercise among middle-aged and elderly Japanese, has not been fully elucidated, and the epidemiological evidence on the association between walking exercise and lipids is limited^16^^,^^17^^)^. This study sought to examine the relation of walking, which people take regularly as an exercise for health, to serum lipids among the middle-aged and elderly in Japan.

## MATERIALS AND METHODS

### Subjects and characteristics

The subject group consisted of 3312 adult Japanese who had undergone a routine health examination at Yukuhashi city for early detection of disease in May and October 1998. Located in the eastern part of Fukuoka Prefecture, Yukuhashi city is a population of about 67,000, and the percentage of the population older than 65 years is 15%.

### Definition of the categories

Walking for exercise was measured by a self-reported questionnaire: Do you regularly exercise for health? and what kind of sport do you take? Smoking status (currently smokes, past smoker, or never smoked), alcohol intake (total abstinence, occasionally drinks, or daily drinker) were also checked by questionnaire. The participants were divided into five groups according to the total weekly minutes spent walking exercise (persons who did not walk as exercise, 1-120, 121-240, 241-360, over 361 minutes per week). Body mass index was calculated by dividing body weight (kg) by body height (m^2^). Total cholesterol, HDL cholesterol, and triglyceride were measured using fasting blood samples. All samples were measured at the same facility. Total cholesterol was measured by Total Cholesterol-HR (Wako Pure Chemical Industry Co., LTD.) according to the COD-DAOS method. HDL cholesterol was measured by cholestest HDL (Daiichi Pure Chemicals Co., LTD., Tokyo, Japan) according to the method of direct assay. Low-density lipoproteins cholesterol (LDL cholesterol) was estimated by the Friedewald equation^18^^,^^19^^)^. The examination period was divided into two seasons, spring (May and July) and autumn (October and November).

### Exclusion

We excluded 69 individuals taking medicine at the time for hyperlipidemia. Individuals who engaged in athletic activities other than walking exercise were excluded (140 swimming, 58 ballgames, 45 keep-fit programs on the radio, 180 unknown). Hence, 808 males and 2012 females took part in this study.

### Statistical analysis

Analyses of variance were performed to calculate the adjusted means for total cholesterol, HDL cholesterol, ratio of total cholesterol to HDL cholesterol, triglyceride, and LDL cholesterol using total walking time categories as the level of a factor. For multivariate procedures, age, smoking status, alcohol intake, body mass index, and season were entered as covariates. Age and body mass index were treated as continuous data, and smoking status, alcohol intake, and seasons were treated as categorical data. Logistic regression analyses adjusted for age, smoking status, alcohol intake, body mass index and season were performed to estimate odds ratios (ORs) and their 95% confidence intervals (CIs) for total cholesterol≧220, HDL cholesterol≦40, the ratio of total cholesterol to HDL cholesterol≧5.5, triglyceride≧150, and LDL cholesterol≧150 taking individuals who did no walking as controls. All analyses were performed with the Statistical Analysis System for personal computers, Release 6.12^20^^)^.

## RESULTS

As shown in table 1, 83% of the men were 60 to 79 years old, while 70% of the women were 50 to 69 years old. Forty-seven percent of the men and 35% of the women walked for health reasons. Among those who walked, the mean walking time per week for men was 283 minute/week. The figure for women was similar.

**Table 1.** Walking time per week by age and sex.

Age	N	persons who walked		

		%^a^	Mean(minute/week)	SD
Male				
-49	29	13.8	237.5	148.9
50-59	68	26.5	220.3	119.1
60-69	434	49.8	301.9	156.0
70-79	237	52.7	261.9	112.8
80-	40	35.0	267.9	119.8
Total	808	46.7	282.8	141.4

Female				
-49	280	24.3	243.9	101.8
50-59	612	39.6	281.5	142.5
60-69	798	39.1	274.2	126.0
70-79	296	30.7	267.4	117.5
80-	26	38.5	198.0	72.1
Total	2012	35.1	271.7	128.3

^a^ Percentage of regular walking in each age group.

As shown in table 2, men who walked were older and less likely to smoke than men who did not walk. Women who walked a great deal (over 241 minute/week) were less likely to smoke. There were no significant differences in other possible confounding factors, such as body mass index, alcohol intake and season, according to walking time in both sexes.

**Table 2.** Characteristics by walking time.

Walking time (minute/week)	N	Age		BMI^a^ (kg/m^2^)		Smoking status (%)	Alcohol intake (%)			Season (%)
				
		Mean	SD	Mean	SD	currently smokes	never	occasionally	everyday	Spring
Male										
no exercise	431	65.6	8.9	23.3	2.8	34.6	27.1	22.0	50.9	48.5
1-120	47	66.1	6.9	22.8	3.0	27.7	28.3	26.1	45.7	42.6
121-240	142	68.0	6.9	23.2	2.9	21.8	24.8	24.1	51.1	57.0
241-360	80	68.8	6.2	23.1	2.5	23.8	21.1	19.7	59.2	48.8
361-	108	66.5	5.9	23.6	2.6	25.9	34.0	13.2	52.8	54.6
P value		0.002		0.44		0.02	0.35			0.27

Female										
no exercise	1305	60.0	9.5	22.9	3.1	4.5	6.1	20.1	73.0	50.7
1-120	62	60.8	9.0	22.8	2.8	4.8	6.9	22.4	4.0	51.6
121-240	296	60.7	8.6	23.2	3.2	3.7	3.8	22.7	10.0	52.4
241-360	188	61.6	8.2	23.0	2.9	0.5	6.5	15.9	11.0	52.7
361-	161	60.8	7.4	23.1	3.0	1.2	4.1	16.9	6.0	60.3
P value		1.61		0.62		0.03	0.50			0.26

^a^ Body mass index

In table 3, total cholesterol was significantly higher for those who walked than those who did not walk. The adjusted means for total cholesterol were 201.9mg/dl in men who walked and 194.9mg/dl in men who did not walk (P<0.01); and 217.6mg/dl in women who walked and 212.9mg/dl in women who did not walk (P<0.01). However, among those who walked, total cholesterol did not increase as walking time increased. HDL cholesterol in men who walked was significantly higher than in men who did not walk (P<0.03). This difference was not observed for the women. There were no significant differences in triglyseride, or the ratio of total cholesterol to HDL cholesterol, according to walking in either men or women. LDL cholesterol, however, was significantly higher for those who walked than those who did not walk.

**Table 3.** Mean levels of lipids by walking time adjusted for age, body mass index, smoking status, alcohol intake and season.

Walking time (minute/week)	N	Total cholesterol (mg/dl)		HDL cholesterol (mg/dl)		TCH/HDL^a^		TG (mg/dl)		LDL cholesterol (mg/dl)	
				
		Mean	SE	Mean	SE	Mean	SE	Mean	SE	Mean	SE
Male											
no exercise	431	194.9	2.0	53.0	0.7	3.9	0.07	115.5	3.8	119.2	1.9
regularly walking	377	201.9	2.1	55.0	0.8	3.9	0.07	111.0	4.1	124.6	2.1
P value		<0.01		0.03		0.61		0.33		0.02	

1-120	47	200.6	5.3	59.1	2.0	3.6	0.16	90.5	12.3	120.3	6.3
121-240	142	200.7	3.2	54.8	1.3	6.9	0.09	115.6	5.6	123.7	2.9
241-360	80	206.3	4.2	54.4	1.6	4.1	0.12	101.8	7.5	130.6	3.8
361-	108	200.8	3.7	56.1	1.5	3.9	0.11	117.6	6.8	122.4	3.4
P value		0.07		0.21		0.24		0.12		0.28	
P for trend		0.58		0.56		0.35		0.30		0.66	

Female											
no exercise	1305	212.9	2.6	62.5	1.0	3.6	0.08	113.0	4.1	128.1	2.5
regularly walking	707	217.6	2.8	63.1	1.0	3.7	0.08	110.8	4.5	133.1	2.7
P value		<0.01		0.30		0.26		0.43		<0.01	

1-120	62	219.9	5.4	60.5	2.6	3.8	0.16	114.0	9.4	133.2	7.1
121-240	296	219.1	3.3	62.8	2.1	3.7	0.10	113.3	5.2	134.6	5.4
241-360	188	213.9	3.8	61.6	2.2	3.7	0.11	115.1	5.8	129.7	5.7
361-	161	221.5	3.9	62.8	2.3	3.7	0.12	100.3	6.0	139.6	5.7
P value		0.02		0.54		0.60		0.09		0.07	
P for trend		0.80		0.69		0.85		0.03		0.27	

^a^ Ratio of total cholesterol to HDL cholesterol

As shown in table 4, men who walked had a higher OR for hypercholesterolemia than men who did not walk (total cholesterol≧220: OR=1.69, 95% CI=1.22-2.35), and women (total cholesterol≧220: OR=1.27,95% CI=1.04-1.53). Walking 121 to 360 minutes per week was associated with an increased OR for hypercholesterolemia in both men (total cholesterol≧220: OR=2.24, 95% CI=1.31-3.83 for 241-360 minutes/week), and women (total cholesterol≧220 mg/dl: OR=1.32, 95% CI=1.00-1.74 for 121-240 minutes/week). For both sexes, ORs for HDL cholesterol≦40 mg/dl did not significantly differ for those who walked and those who did not. This was also true of ORs for the ratio of total cholesterol to HDL cholesterol≧5.5. Women only who walked more than 360 minute in a week decreased the OR for triglyceride≧150 mg/dl. The OR for LDL≧150 mg/dl was higher in women who walked than in those who did not walk (OR=1.23, 95% CI=1.00-1.52).

**Table 4.** Odds ratio and confidence intervals adjusted for age, body mass index, smoking status, alcohol intake and season by walking time.

Walking time (minute/week)	N	Total cholesterol≧220 (mg/dl)			HDL cholesterol≦40 (mg/dl)			TCH/HDL^a^≧5.5			TG≧150 (mg/dl)			LDL cholesterol≧150 (mg/dl)		
				
		n	OR	95%CI	n	OR	95%CI	n	OR	95%CI	n	OR	95%CI	n	OR	95%CI
Male																
no exercise	431	92	1.00		67	1.00		34	1.00		95	1.00		73	1.00	
regularly walking	377	115	1.69	1.22-2.35	44	0.78	0.51-1.20	23	1.05	0.60-1.84	65	0.87	0.60-1.26	76	1.34	0.91-1.96

no exercise	431	92	1.00		67	1.00		34	1.00		95	1.00		73	1.00	
1-120	47	14	1.46	0.73-2.93	3	0.45	0.13-1.53	2	0.60	0.14-2.64	2	0.30	0.07-1.32	3	0.62	0.18-2.17
121-240	142	39	1.36	0.87-2.14	12	0.51	0.26-1.01	8	0.74	0.32-1.68	32	1.14	0.71-1.82	30	1.29	0.78-2.13
241-360	80	30	2.24	1.31-3.83	13	1.36	0.69-2.70	5	1.06	0.39-2.89	9	0.53	0.25-1.12	23	2.20	1.22-3.97
361-	108	32	1.51	0.92-2.47	16	1.00	0.54-1.86	8	0.99	0.83-2.28	22	0.97	0.56-1.68	20	1.12	0.63-2.01

Female																
no exercise	1305	592	1.00		69	1.00		66	1.00		185	1.00		417	1.00	
regularly walking	707	367	1.27	1.04-1.53	45	1.12	0.74-1.68	49	1.35	0.91-2.02	92	0.90	0.67-1.20	260	1.23	1.00-1.52

no exercise	1305	592	1.00		69	1.00		66	1.00		185	1.00		417	1.00	
1-120	62	31	1.55	0.88-2.72	4	1.46	0.51-4.18	2	0.75	0.18-3.20	4	0.74	0.26-2.16	13	1.01	0.50-2.04
121-240	296	153	1.32	1.00-1.74	16	0.99	0.53-1.82	23	1.59	0.94-2.71	47	1.10	0.75-1.62	113	1.26	0.95-1.68
241-360	188	91	0.99	0.71-1.38	14	1.19	0.59-2.38	15	1.69	0.90-3.19	29	1.00	0.62-1.63	60	0.88	0.62-1.26
361-	161	92	1.51	1.07-2.15	11	1.44	0.74-2.81	9	1.31	0.63-2.71	12	0.50	0.27-0.94	74	1.72	1.22-2.43

^a^ Ratio of total cholesterol to HDL cholesterol

## DISCUSSION

This study investigated the relation of walking exercise to serum lipids for middle-aged and elderly individuals living in a Japanese community. Waking is the most common form of exercise for health among middle-aged and elderly Japanese. We found higher total cholesterol levels, HDL cholesterol, and LDL cholesterol in individuals who walked, suggesting that walking exercise may not achieve the beneficial effect on lipids profiles among Japanese middle-aged/older men and women. Even though, physical activity is well known to decrease triglyceride^21^^)^, there was no difference in the level of triglyceride between those who took walking exercise and those who did not in this study. The possible explanation of these results is that walking exercise among middle-aged and elderly person is insufficient intense to affect on lipid profiels. Alternatively, the effect of walking and physical activity is more significant when the effect of exercise on lipoprotein subfractions and apolipoprotein levels are taken into account^21^^)^. Dramatic differences can be documented in concentrations of cardioprotective HDL subset2 and atherogenic small, dense LDL subset6 levels in relation to the individual status of physical activity and body composition.

Another study achieved similar results: Hakim et al. showed higher levels of total cholesterol in elderly Japanese men who had emigrated and their descendants who walked for exercise^6^^)^. However, the majority of observational studies have not demonstrated any significant associations between walking or similar low intensity sports activities and levels of total cholesterol^8^^,^^9^^,^^12^^,^^13^^,^^16^^,^^22^^-^^24^^)^. Similarly, the absence of association between them has been indicated by many intervention studies^12^^,^^13^^,^^15^^,^^25^^)^. In well-controlled studies, findings were inconsistent; some found a decrease in total cholesterol levels among those who walked regularly, while others did not^10^^,^^12^^,^^13^^,^^15^^,^^26^^)^. When interpreting these inconsistent results, we need to consider that total cholesterol levels are significantly affected by numerous factors, including diet, calorie intake, coffee consumption, alcohol consumption, cigarette smoking, medication, body weight, body composition, levels of physical activity, and so on^12^^,^^13^^,^^15^^)^. In our study, we found higher levels of total cholesterol among those who walked - even after adjusting for age, body mass index, alcohol consumption, cigarette smoking, and season. However, we did not obtain information on diet composition, which may influence total cholesterol levels more than any other factor. If our subjects who engaged in walking consumed more food containing cholesterol than those who did not, this would have skewed our findings.

In our study, we found higher levels of HDL cholesterol in men who walked, a finding consistent with previous studies^8^^,^^9^^,^^12^^-^^15^^)^. However, such findings were not observed in women. Possible explanations for this difference include the effects of hormonal status on lipid metabolism. Menopausal women often experience reduced HDL cholesterol levels^15^^,^^27^^-^^29^^)^. In our study, we did not obtain information on hormonal status, such as menstrual status, pregnancy history, or phase of menstrual cycle. However, even studies with adjustments for sex hormonal status (e.g., menopausal status, hormonal replacement therapy, and oral contraceptive use) have reported inconsistent relationships between walking and lipoprotein levels^15^^)^.

We found no significant differences in the ratio of total cholesterol to HDL cholesterol between individuals who walked and those who did not, even though total cholesterol levels in individuals who walked were higher than in individuals who did not walk. This suggests that elevated total cholesterol levels accompany elevated levels of HDL cholesterol.

Our study included certain potential biases and problems. First, the present study assessed walking but not total physical activity, including physical activity at work, odd jobs, and hobbies. Such activities may result in the present association. Individuals who walked may be more physically active in other settings than those who not. If so, our estimates of the effects of walking may be inflated. Second, although we excluded individuals taking medicine at the time, individuals who have clinical and subclinical hypercholesterolemia may engage in higher levels of walking to improve their lipid profiles. We cannot discount such explanations from the present cross-sectional study. Third, we did not obtain information on distance, intensity, and frequency of walking, aspects that have been used as indicators of walking activity in previous studies^5^^-^^8^^,^^30^^)^. Instead, we simply used the total time spent per week walking. We believe this measure is likely to be equivalent to the distance walked or energy thus consumed, since the total amount of time walked per week was highly correlated with the energy expended when walking^9^^)^, and because intensity of walking might be similar among the middle-aged and elderly who walked regularly.

In conclusion, we found that walking was associated with higher total and LDL cholesterol levels in both sexes and higher levels of HDL cholesterol in men. However, it remains unclear whether walking exercise among general population provides beneficial effects on lipid profiles. Additional studies are needed to examine the association of walking with lipid profiles, and more generally to identify the effects of walking on general health. Such studies should account for dietary factors, or examine trends in the intensity of walking and lipid profiles.
